# Modulation of Ca^2+^-induced Ca^2+^ release by ubiquitin protein ligase E3 component n-recognin UBR3 and 6 in cardiac myocytes

**DOI:** 10.1080/19336950.2020.1824957

**Published:** 2020-09-29

**Authors:** Xiu-E Ma, Bei Liu, Chun-Xia Zhao

**Affiliations:** a Key Laboratory of Arrhythmias of the Ministry of Education of China, East Hospital, Tongji University School of Medicine, Shanghai, China; b Department of Cardiology, Shanghai General Hospital, School of Medicine, Shanghai Jiaotong University, Shanghai, China

**Keywords:** UBR, Ca^2+^-induced Ca^2+^ release, Ca_v_1.2 channel

## Abstract

Ca^2+^-induced Ca^2+^ release (CICR) from sarcoplasmic reticulum is a finely tuned process responsible for cardiac excitation and contraction. The ubiquitin–proteasome system (UPS) as a major degradative system plays a crucial role in the maintenance of Ca^2+^ homeostasis. The E3 component N-recognin (UBR) subfamily is a part of the UPS; however, the role of UBR in regulating cardiac CICR is unknown. In the present study, we found that among the UBR family, single knockdown of UBR3 or UBR6 significantly elevated the amplitude of sarcoplasmic reticulum Ca^2+^ release without affecting Ca^2+^ transient decay time in neonatal rat ventricular myocytes. The protein expression of alpha 1 C subunit of L-type voltage-dependent Ca^2+^ channel (Ca_v_1.2) was increased after UBR3/6 knockdown, whereas the protein levels of RyR2, SERCA2a, and PLB remained unchanged. In line with the increase in Ca_v_1.2 proteins, the UBR3/6 knockdown enhanced the current of Ca_v_1.2 channels. Furthermore, the increase in Ca_v_1.2 proteins caused by UBR3/6 reduction was not counteracted by a protein biosynthesis inhibitor, cycloheximide, suggesting a degradative regulation of UBR3/6 on Ca_v_1.2 channels. Our results indicate that UBR3/6 modulates cardiac CICR via targeting Ca_v_1.2 protein degradation.

## Graphical abstract

**Figure:**
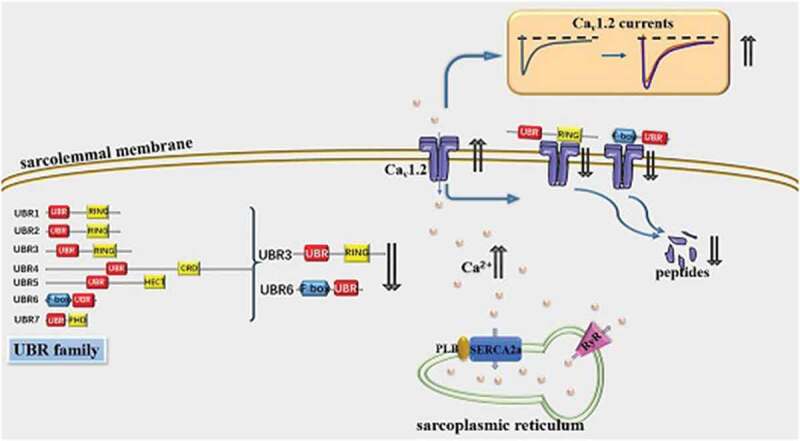

## Introduction

The cardiac Ca^2+^-induced Ca^2+^ release (CICR) is a significant process as calcium influx from extracellular space activates Ca^2+^ release from intracellular Ca^2+^ stores[1]. This process depends on the integration of multiple sarcolemmal and sarcoplasmic reticulum membrane proteins. It is triggered by the L-type Ca^2+^ influx mainly through Ca_v_1.2 channels in the sarcolemmal membrane, and amplified by Ca^2+^ release through the sarcoplasmic reticulum (SR) ryanodine receptors 2 (RyR2). Through the CICR process, calcium transients are generated. The increased cytoplasmic calcium binds to Troponin C, moving the tropomyosin complex off the actin binding site and allowing the myosin to bind the actin filament. Through ATP hydrolysis, the myosin head pulls the actin filament toward the center of the sarcomere. Then, a calcium ion pump sarcoplasmic/endoplasmic reticulum Ca^2+^ ATPase 2a (SERCA2a), which is under the precise control of phospholamban (PLB), transfers most of the Ca^2+^ in the cytoplasm back into the SR, and the muscle fiber relaxes [2,3].

The ubiquitin–proteasome system (UPS) is a cascade reaction consisting of ubiquitin (Ub), E1 (Ub-activating enzyme), E2 (Ub-conjugating enzyme), E3 (Ub-protein ligase) and proteasome. Ub is catalyzed by E1 in an ATP-dependent manner, and E2 accepts the activated Ub on cysteine. E3 mediates the transfer of the activated Ub from E2 to target protein[4]. Finally, the ubiquitinated protein is degraded by the 26S proteasome[5]. UPS plays an important role in regulating the degradation of many kinds of membrane proteins, such as receptor tyrosine kinases (RTK), the general amino acid permease Gap1p, connexin 43, tyrosine-phosphorylated E-cadherin and certain ion channels [6,7]. One of its subfamilies termed E3 component N-recognin (UBR) contains at least seven UBR box-containing proteins (UBR1-UBR7) [8,9]. The UBR family perform various functions in cardiovascular system, such as proliferation of cardiomyocytes and cardiovascular development [10,11]. Accumulative data show that the polypeptide ubiquitin is a key down-regulator of many plasma membrane proteins[12]. However, whether UBRs have effects on cardiac CICR through regulating the calcium-handling proteins involved in CICR is still elusive.

The aim of this study is to elucidate the implication of the modulation of ubiquitin ligase UBR on cardiac calcium-handling proteins. We studied CICR by measuring Ca^2+^ transients in UBR-knockdown rat cardiomyocytes and found that UBR3/6 changed CICR. Further analysis revealed that the knockdown of UBR3 and UBR6, significantly increased the level of Ca_v_1.2 channel proteins, not other sarcolemmal or SR membrane proteins involved in CICR. The function of Ca_v_1.2 channel was also enhanced in response to UBR3/6 knockdown. The employment of protein synthesis inhibitor cycloheximide showed that UBR3/6-knockdown-mediated increases of Ca_v_1.2 proteins were not derived from de novo synthesis.

## Materials and methods

### Preparation of primary neonatal rat ventricular myocytes

All of the animal experiments were approved by the Animal Experiments Committee of Tongji University and conformed to the Guide for the Care and Use of Laboratory Animals established the US National Institutes of Health.

Neonatal rat ventricular myocytes (NRVMs) from the ventricles of 1–2-day-old Sprague–Dawley neonatal rats were isolated as previously described[13]. The hearts from neonatal rats were excised, minced, and digested in PBS solution containing trypsin (0.25%), collagenase (0.1%), and DNAase (1%) for 5 min at 37^◦^C. The same procedure was repeated five times. The isolated cells were collected, cultured in a CO_2_ incubator, and purified by differential adhesion for 2 h.

### Cell culture and transfection

The NRVMs were maintained in Dulbecco’s Modified Eagle’s Medium (DMEM) supplemented with 10% fetal calf serum (Gibco, BRL Co. Ltd., USA) and 1% penicillin and streptomycin in a humidified incubator at 37°C with 5% CO2. 48 h later, the NRVMs were transfected with siRNAs for 48 h and collected for downstream assay.

### RNA interference

Rat UBR1-7 were knocked down by specific small interference RNAs (siRNAs) which were synthesized by Jima Biotechnology Co., Ltd. (Shanghai, China). A 21-mer scrambled double-stranded RNA was used as the negative control. All of the siRNA sequences are listed in Table 1. Primary neonatal rat ventricular myocytes were transfected with siRNA and Lipofectamine RNAiMAX Reagent (Invitrogen, Carlsbad, CA, USA) according to the manufacturer’s protocols.

**Table 1.** All of the siRNA sequences used in this study.

Species	Target gene symbol	Sequence (5ʹ–3ʹ)
Rat	UBR1	S-GGCCCGACAUCUUAUUGAATT
		A-UCAAUAAGAUGUCGGGCCTT
	UBR2	S-GCGCCACAGAUGAAAUCAATT
		A-UUGAUUUCAUCUGUGGCGCTT
	UBR3	S-GCGGCACUUUAUAAAUUAUTT
		A-AUAAUUUAUAAAGUGCCGCTT
	UBR4	S-CUCCACCACAGAUGAAGAATT
		A-UUCUUCAUCUGUGGUGGAGTT
	UBR5	S-GGGCCUUAUUCCUAAGUAUTT
		A-AUACUUAGGAAUAAGGCCCTT
	UBR6	S-GUCCAAUCCUUGUACAUUATT
		A-UAAUGUACAAGGAUUGGACTT
	UBR7	S-GACUGAACUUAAGGAUUAUTT
		A-AUAAUCCUUAAGUUCAGUCTT
	Negative control	S-UUCUCCGAACGUGUCACGUTT
		A-ACGUGACACGUUCGGAGAATT

S: sense; A: antisense

### Immunofluorescence

Adherent cells were fixed with 4% paraformaldehyde for 15 min, permeabilized with 0.1% Triton X-100 and then blocked with 1% BSA for 1 hr. After incubation with anti-α-actin (Sigma) as primary antibody overnight at 4°C, FITC-conjugated goat anti rabbit IgG (Abcam) was used as secondary antibody. 6-diamidino-2-phenylindole (DAPI) was used for nuclear counterstaining. The slides were photographed using fluorescence microscopy (Leica, Germany). The dilution concentration of the primary antibodies was 1:10 to 1:100, and the secondary antibodies at a dilution of 1:200.

### Determination of [Ca^2+^]_i_

Intracellular calcium was measured using a dual-excitation fluorescence photomultiplier system (Ion Optix) as described [14]. NRVMs was loaded with Fura-2-AM (1 μmol/l) for 30 min in the dark and then washed with PBS prior to imaging. Fura-2-AM-loaded cells were monitored by a fluorescent monitoring system with wavelength settings of 340 and 380 nm for excitation and between 480 and 520 nm for emission in a photomultiplier tube. Intracellular calcium concentration was analyzed as the ratio of fluorescence intensity (F340/F380). The time of 50% decay (T50) was chosen as the time interval from the peak to the time at which the signal had decayed to 50% of peak value.

### Western blot analysis

The cells were lysed using RIPA lysis buffer (150 mM NaCl, 50 mM Tris–HCl (pH 7.4), 1% sodium deoxycholate, 1% NP-40, 1 mM PMSF and 1 mM EDTA) at 4°C for 20 min. The proteins were separated by SDS-PAGE gel (Invitrogen), and transferred to polyvinylidene fluoride membranes (Invitrogen). The membranes were incubated overnight at 4°C with the appropriate primary antibodies followed by the peroxidase-conjugated secondary antibody for 1 hr at room temperature. Then the immunoblots were visualized using chemiluminescence reagents. The primary antibodies included anti-UBR3 antibodies (Santa Cruz Biotechnology), anti-UBR6 antibodies (Santa Cruz Biotechnology), anti-Cav1.2 antibodies (Alomone Labs), anti-RYR2 antibodies (Abcam), anti-SERCA2a antibodies (Abcam), anti-PLB antibodies (Cell Signaling), and anti-GAPDH antibodies (Cell Signaling). The dilution concentration of the primary antibodies was 1:200 to 1:1000, and the secondary antibodies at a dilution of 1:1000. Results are representative of at least three independent experiments.

### Patch-clamp techniques

Standard voltage clamp technique was used to record the cardiac L-type Ca^2+^ current as previously described [14]. After 2 days of cell culture, whole-cell patch-clamp recordings were performed at room temperature using an EPC-10 amplifier and pulse software (HEKA, Lambrecht, Germany). Electrophysiological properties in single cardiomyocytes were acquired from healthy NRVMs. The extracellular solution (pH 7.4, titrated with CsOH) contained (in mM): TWCL 136, CsCl 5.4, CaCl_2_ 2, MgCl_2_ 0.8, dextrose 10, and HEPES 10. The intracellular solution (pH 7.2, titrated with CsOH) contained (in mM): CsCl 130, GTP 0.1, EGTA 10, Na^−^ phosphocreatine 5, MgCl_2_ 1, MgATP 5, and HEPES 10.

### Cycloheximide blocking assay

This was accomplished by treating UBR3 or UBR6 knockdown cells with or without cycloheximide to inhibit protein translation. The NRVMs were treated with cycloheximide (100 µg/ml) (CHX; 10 μM; Calbiochem, San Diego, CA, USA) for 0, 4, 8, and 12 h, following UBR3 or UBR6 siRNAs treatment for 24 h. Total proteins were extracted from the cell samples, and Western blot was performed to analyze Ca_v_1.2 channel protein levels.

### Statistical analysis

All data were analyzed using SPSS 13.0 software (SPSS Inc., Cary, NC, USA) and presented as means ± SEM. In all experiments, unpaired Student’s t-test or one-way ANOVA was used to compare experimental groups with their appropriate controls. p < 0.05 was considered statistically significant. Ca^2+^ transient and voltage-clamp data were analyzed using Pulsefit (HEKA) and Origin 7.5 (OriginLab, Northampton, MA, USA). Each experiment was repeated at least three times.

## Results

### Knockdown of UBR3 or UBR6 increases Ca^2+^ transient amplitude in cardiomyocytes

**Figure 1.**
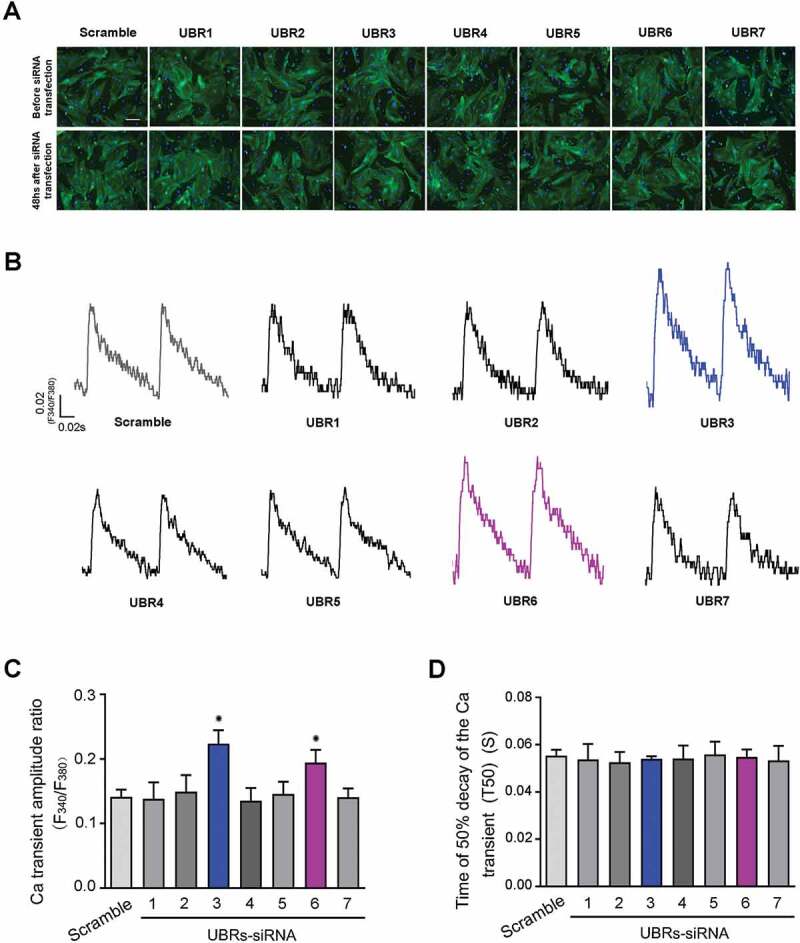
Ca^2+^ transient amplitude in cardiomyocytes.

In order to explore the effects of UBRs on Ca^2+^ transient in cardiomyocytes, the UBR1-7 siRNAs were individually transfected into NRVMs. After 48 hours, the knockdown efficiency of UBR1-7 siRNA was tested by RT-PCR. The cross-inhibition and inhibition efficiency of UBR1-7 siRNAs are listed in Table 2. The overall morphology of the isolated and transfected NRVMs was indicated by immunofluorescence staining with α-actin, the representative fluorescence results are shown in Figure 1A. Intracellular Ca^2+^ transient amplitude and Ca^2+^ transient decay time was measured in Fura-2/AM loaded NRVMs under electric field stimulation (Figure 1B). As shown in Figure 1C, individual knockdown of UBR3 or UBR6, but not other UBR members, significantly increased the Ca^2+^ transient amplitude in F_340_/F_380_ ratio from (0.141 ± 0.006) to (0.224 ± 0.031) and (0.192 ± 0.017) respectively. However, the Ca^2+^ transient decay time (T50) was not significantly changed by UBRs knockdown (Figure 1D). These results suggest that UBR3 and UBR6 of UBR family may play an important role in the regulation of CICR.

**Table 2.** The inhibition efficiency of UBR siRNAs on UBR members.

	UBR1	UBR2	UBR3	UBR4	UBR5	UBR6	UBR7
SiRNA of UBR1	>70%*	<25%	35–25%	<25%	<25%	<25%	<25%
SiRNA of UBR2	<25%	>70%*	35–25%	35–25%	<25%	<25%	<25%
SiRNA of UBR3	35–25%	35–25%	50–70%*	<25%	35–25%	<25%	<25%
SiRNA of UBR4	<25%	<25%	<25%	35–50%*	<25%	<25%	<25%
SiRNA of UBR5	<25%	<25%	<25%	<25%	>70%*	<25%	<25%
SiRNA of UBR6	35–25%	<25%	35–25%	35–25%	<25%	>70%*	35–25%
SiRNA of UBR7	35–25%	35–25%	35–25%	35–25%	35–25%	<25%	>70%*
SiRNA of Negative control	<25%	<25%	<25%	<25%	<25%	<25%	<25%

n = 3 *p < 0.01

(A) Immunofluorescence analysis of the isolated NRVMs before and after siRNA transfection. NRVMs were stained for α-acting (green). DAPI (blue) was used to stain nuclei. Scale bars, 100 μm. (B) Typical recordings of cardiac Ca transient amplitude ratio (F340/F380). (C) Analysis of cardiac Ca transient amplitude ratio (F340/F380) in UBRs knockdown NRVMs (*n* = 3, *p < 0.01). (D) Analysis of time to 50% decay of the Ca transient (T50) in UBRs knockdown NRVMs (*n* = 3, *p < 0.01).

### Knockdown of UBR3 and UBR6 elevates the cardiac Ca_v_1.2 channels protein expression

**Figure 2.**
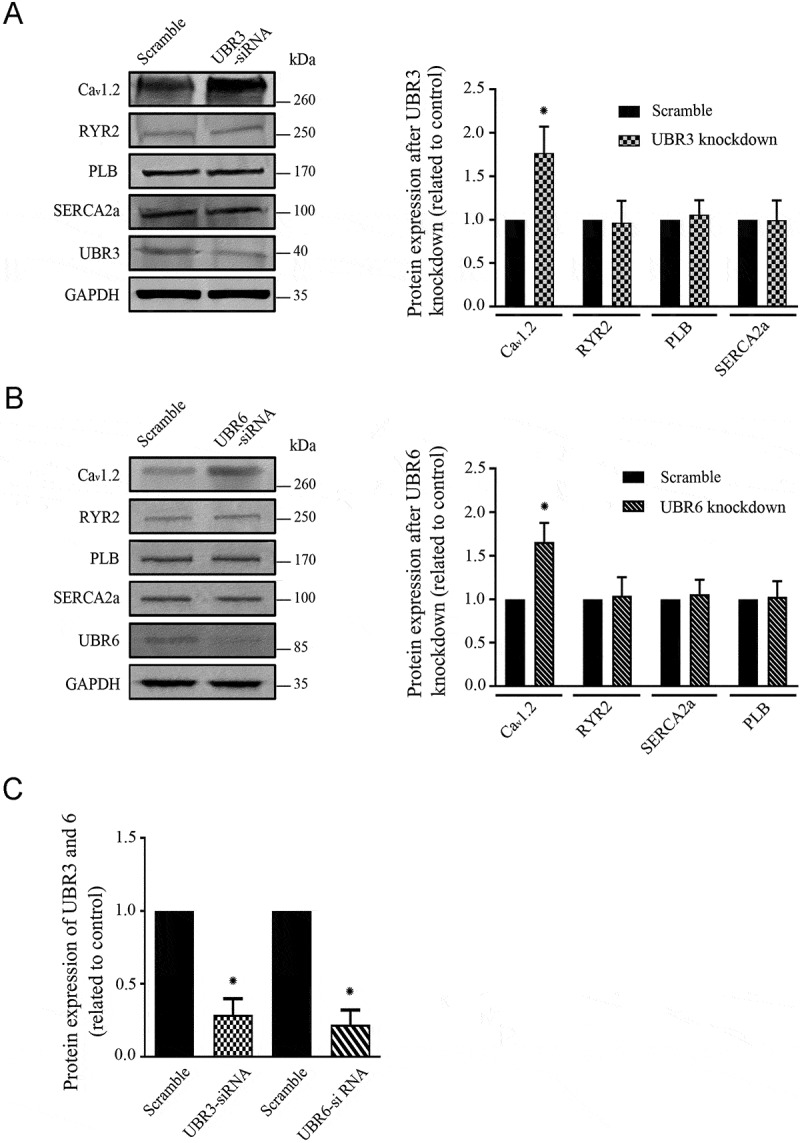
Protein expression of the Ca^2+^-handling proteins on the sarcolemmal and sarcoplasmic reticulum membranes in UBR3/6 knockdown NRVMs.

To further determine the CICR-associated proteins regulated by UBR3/6, the levels of Ca^2+^ handling proteins including RyR2, SERCA2a, PLB, and Ca_v_1.2 channels were examined in cultured NRVMs following 24-h treatment with control and UBR3/6 siRNA (Figure 2). The results showed that the expression of UBR3 and UBR6 significantly reduced by (71.33 ± 11.04)% and (87.83 ± 10.06)%, respectively. Meanwhile, following knockdown of either UBR3 or UBR6, the expression of Ca_v_1.2 channel protein was significantly increased by (177 ± 31)% and (166 ± 22)%, respectively. However, the expression levels of RyR2, SERCA2a, and PLB were unchanged. These results suggest that UBR3 and UBR6 modulate CICR through targeting Ca_v_1.2 channel proteins.

(A) (B) *Left*: A typical example of western blot analysis showed the expression of Ca^2+^-handling proteins on the sarcolemmal and sarcoplasmic reticulum membranes in UBR3/6 knockdown NRVMs. *Right*: The pooled and quantified western blot data (*n* = 3, *p < 0.01). (C) The pooled data for the knockdown efficiency of UBR3 and UBR6 siRNAs in NRVMs (*n* = 3, *p < 0.01).

### Reduction of UBR3 and UBR6 enhances the current of Ca_v_1.2 channels

**Figure 3.**
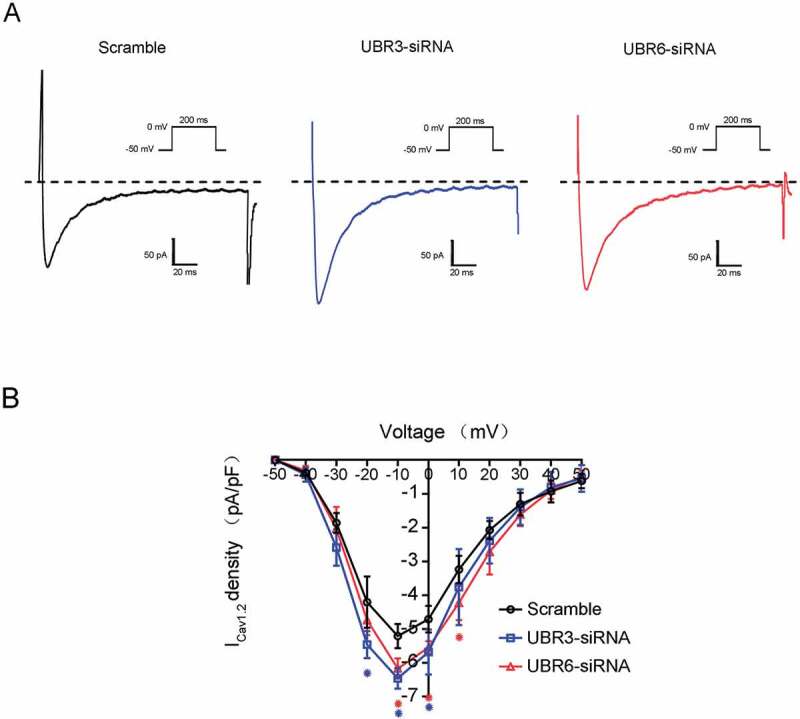
Effects of UBR3/6 knockdown on Ca_v_1.2 channel currents.

Based on the above results, we further examined whether the knockdown of UBR3 or UBR6 could enhance the function of Ca_v_1.2 channels. As shown in the current–voltage (I–V) curves (Figure 3), the peak current density of Ca_v_1.2 channels during the depolarizing step from – 50 to +50 mV in UBR3 or UBR6 knockdown NRVMs was significantly increased (p < 0.01). The peak current density of the Ca^2+^ channels in UBR3 knockdown cells increased from (−5.21 ± 0.36) to (−6.46 ± 0.31) pA/pF (n > 10; p < 0.05). The peak current density generated by the Ca^2+^ channels in UBR6 knockdown cells increased to (−6.17 ± 0.3) pA/pF (n > 10; p < 0.05). These results suggest that the downregulation of UBR3 and UBR6 enhances the activity of Ca_v_1.2 channels.

(A) Representative tracings of Ca_v_1.2 currents from NRVMs. (B) Current-voltage (I–V) relationship of transient I_Ca_ from NRVMs (*n* > 10, *p < 0.01). The current traces were recorded at V_m_ in the range of −50 to +50 mV.

### UBR3 and UBR6 regulate the degradation of Ca_v_1.2 channel proteins

**Figure 4.**
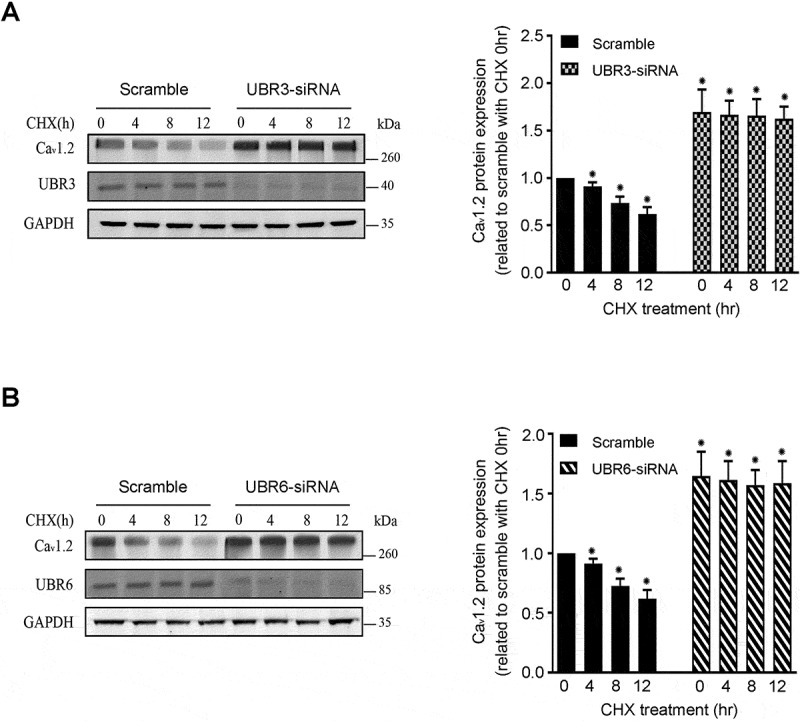
Cycloheximide blocking assay of Ca_v_1.2 channels in UBR3/6 knockdown NRVMs. (A) Effect of cycloheximide (CHX) on Ca_v_1.2 channel expression in UBR3 knockdown NRVMs. NRVMs were transfected with or without UBR3 siRNA for 24 h, then treated with CHX (100 µg/ml). Aliquots of the cells were collected at 0, 4, 8, and 12 h after CHX treatment for western blot analysis. A typical example of a western blot analysis (left panel) and the summarized data (right panel) are shown. GAPDH served as a loading control (*n* = 3, *p < 0.01). Following the treatment of CHX, the Ca_v_1.2 protein level showed a time-dependent decrease. The increase in Ca_v_1.2 protein in UBR3 knockdown cells was not counteracted. (B) Effect of CHX on Ca_v_1.2 channel expression in UBR6 knockdown NRVMs. NRVMs were transfected with or without UBR6 siRNA for 24 h then treated with CHX (100 µg/ml). Aliquots of the cells were collected at 0, 4, 8, and 12 h after CHX treatment for western blot analysis. A typical example of a western blot analysis (left panel) and the summarized data (right panel) are provided. GAPDH served as a loading control (*n* = 3, *p < 0.01). Following the treatment of CHX, the Ca_v_1.2 protein level showed a time-dependent decrease. The UBR6 reduction-induced increase in Ca_v_1.2 protein was not counteracted.

To examine whether the increased expression of Ca_v_1.2 channels is derived from de novo synthesis in NRVMs treated with UBR3 and UBR6 siRNA, we employed CHX, a pan inhibitor of protein synthesis that inhibits ribosome translocation. CHX treatment resulted in time-dependent decreases of endogenous Ca_v_1.2 levels in normal cells. In contrast, CHX treatment did not counteract the increases in Ca_v_1.2 proteins caused by UBR3 and UBR6 knockdown (Figure 4). These results suggest that the upregulation of Ca_v_1.2 proteins caused by UBR3 and UBR6 knockdown is independent of de novo synthesis. It is more likely that UBR3 and UBR6 knockdown reduced the degradation of Ca_v_1.2 channels.

## Discussion

In the present study, we investigated the effects of UBR isoforms on cardiac CICR. We found that within the seven members of the UBR family, only UBR3 and UBR6 had effects on the amplitude of sarcoplasmic reticulum Ca^2+^ release. The UBR3 and 6-mediated regulation of CICR was associated with the degradation of Ca_v_1.2 channel proteins but not other Ca^2+^ handling proteins. Furthermore, knockdown of UBR3/6 enhanced the opening of Ca_v_1.2 channel. Overall, we identified a new regulatory function of UBR3 and UBR6 in cardiac CICR by the degradation of Ca_v_1.2 channel protein.

The balance of Ca^2+^-handling proteins, located on the sarcolemmal and SR membranes, is necessary for maintaining the normal function of cardiac CICR. It is reported that Ca_v_1.2 channels can be regulated by a subtype of E3 ligases, neuronal precursor cell-expressed developmentally down-regulated 4–1 (NEDD4-1), and Ca_v_β-free Ca_v_1.2 channels can be ubiquitinated by E3 ubiquitin-protein ligase complex slx8-rfp subunit (RFP2) and degraded by the proteasome [16,17]. Calpain can activate proteasome to degrade RyR2[18]. Overexpression of HAX-1 led to SERCA2 down-regulation in a proteasome-dependent manner[19]. The above evidences suggest that many CICR-related sarcolemmal and SR membrane proteins are ubiquitinated and degraded by the proteasome, a process regulated by ubiquitin proteasome system (UPS)[20]. There are hundreds of subtypes of E3 ligases in UPS that can specifically recognize the target proteins to ensure the ubiquitination reaction[21]. The UBR family is a unique class of E3 ligases that recognize N-degrons or structurally related determinants for ubiquitin-dependent proteolysis and perhaps other processes as well[22]. It consists of seven members and plays a key role in ubiquitination. Here our research showed that UBR3 and UBR6 of the UBR family have selective effects on Ca^2+^ transient amplitude. We further found that in the Ca^2+^-handling proteins located on the sarcolemmal and SR membranes, the knockdown of UBR3/6 increased the protein level of Ca_v_1.2 channels, but not RyR2, SERCA2a, or PLB proteins, suggesting that Ca_v_1.2 channel is the key target in the modulation of UBR3 and UBR6 on cardiac CICR. Based on these results, we speculate that UBR3 and UBR6 are likely to be new modulators of CICR in cardiomyocytes.

Ca_v_1.2 channel is a major channel that mediates Ca^2+^ influx from extracellular space into the cytoplasm of cardiomyocytes, which triggers Ca^2+^ release from SR through RyR2 channels and generates CICR[23]. Ca_v_1.2 channel, as an important kind of ion channel in cardiomyocytes, is also crucial for the physiological function of heart[24]. Abnormalities of the Ca_v_1.2 channel are associated with a variety of cardiac disorders, for example, arrhythmia[25], heart failure [26,27], Brugada syndrome[28], and cardiac hypertrophy[29]. In recent studies, Ca_v_1.2 was reported to be ubiquitinated by RFP2 and degraded at the proteasome[16]. Meanwhile, Nedd4-1 can promote the sorting of newly synthesized Ca_v_ channels for degradation by both the proteasome and the lysosome[17]. These clues implicate that Ca_v_1.2 channels can be degraded by different kinds of E3 ubiquitin-protein ligases. UBR3 and UBR6-mediated regulation could be a novel mechanism of the regulation of Ca_v_1.2 channels. In the present studies, we observed that the knockdown of both UBR3 and UBR6 increased not only the level of Ca_v_1.2 channel proteins, but also its peak current density. That is to say, UBR3 and UBR6 can modulate Ca_v_1.2 channels, and affect the electrophysiological properties of the whole heart. The effect of UBR3/6 on Ca_v_1.2 channels may be an essential precondition for normal cardiac physiology. Our results showed that only UBR3 and UBR6 facilitated the degradation of Ca_v_1.2 channels among the UBR family. Although UBR3 and UBR6 belong to the UBR family, unlike other typical N-recognins, they are not bound to the known N-end rule substrates (N-degrons) of UBR1 and UBR2 [22,30], so they are two special members in this family. It is reported that UBR3 and UBR6 have their distinctive functional domains except the UBR-box of UBR family[31]. UBR3 contains a RING ubiquitylation domain, and UBR6 has the F-box motif[22]. So, we speculate that UBR3 and UBR6 play the same role in the regulation of Ca_v_1.2 channel via their distinctive functional domains in different physiological and pathologic conditions.

In the present study, we found the regulation of UBR3/6 on Ca_v_1.2 in NRVMs; nevertheless, the mechanism of CICR shows developmental changes in rat hearts. In NRVMs, the elevation of cytosolic calcium concentration upon depolarization is mainly mediated by sarcolemmal Ca_v_1.2-mediated calcium influx, whereas, calcium release from the sarcoplasmic reticulum contributes to the majority of calcium elevation in adult cardiomyocytes upon membrane depolarization. This indicates an important functional transition of the L-type Ca^2+^ channels from Ca^2+^ entry path to a predominantly Ca^2+^ trigger [32,33]. It seems conservative for ubiquitinylation-dependent control of Ca_v_1.2 protein homeostasis, which implicates the potency of UBR in controlling Ca_v_1.2 protein expression in adult cardiomyocytes [34,35]. However, the conclusions need to be verified in adult cardiomyocytes in further study. In summary, our work demonstrated that UBR3/6 are involved in the regulation of CICR by reducing the protein levels and the opening of Ca_v_1.2 channels. This discovery has novel pathophysiological implication in heart diseases associated with CICR. UBR3 and UBR6 might become potential targets for therapeutic intervention in diseases associated with electrical and contractile dysfunction.
